# Three *Drosophila* Hox Complex microRNAs Do Not Have Major Effects on Expression of Evolutionarily Conserved Hox Gene Targets during Embryogenesis

**DOI:** 10.1371/journal.pone.0031365

**Published:** 2012-02-29

**Authors:** Derek Lemons, Adam Paré, William McGinnis

**Affiliations:** Section of Cellular and Developmental Biology, Division of Biological Sciences, University of California, San Diego, La Jolla, California, United States of America; University of Massachusetts Medical School, United States of America

## Abstract

The discovery of microRNAs has resulted in a major expansion of the number of molecules known to be involved in gene regulation. Elucidating the functions of animal microRNAs has posed a significant challenge as their target interactions with messenger RNAs do not adhere to simple rules. Of the thousands of known animal microRNAs, relatively few microRNA:messenger RNA regulatory interactions have been biologically validated in an normal organismal context. Here we present evidence that three microRNAs from the Hox complex in *Drosophila* (miR-10-5p, miR-10-3p, miR-iab-4-5p) do not have significant effects during embryogenesis on the expression of Hox genes that contain high confidence microRNAs target sites in the 3′ untranslated regions of their messenger RNAs. This is significant, in that it suggests that many predicted microRNA-target interactions may not be biologically relevant, or that the outcomes of these interactions may be so subtle that mutants may only show phenotypes in specific contexts, such as in environmental stress conditions, or in combinations with other microRNA mutations.

## Introduction

MicroRNAs (miRNAs) are a recently discovered class of biological molecules that have greatly expanded our knowledge concerning post-transcriptional regulation of gene expression. Since the discovery of these small RNA regulatory molecules, many of the proteins involved in their biogenesis, transport, and regulatory functions have been characterized [1]. While much certainly remains to be elucidated concerning the protein components of the miRNA pathway; the larger ‘black box’ is that of target interactions between miRNAs and messenger RNAs (mRNAs).

The first major discoveries in the field of miRNA research were made through careful analyses of genetic mutations in the nematode *C. elegans* with obvious phenotypes [2]–[4]. These studies gave rise to the first ideas of how miRNAs interact with their target genes. Subsequent systematic mutational analyses of established miRNA target sites as well as synthetic miRNA targets provided researchers with the first set of target pairing ‘rules’ for animal miRNAs [5]–[8]. These aided in the development of a number of computational algorithms which allow researchers to predict potential mRNA targets for a given miRNA, or conversely, potential miRNAs which target a specific mRNA [9]. However, due to the relative lack of functional data for validated miRNA:mRNA target interactions, these algorithms were largely based on small training sets in combination with the aforementioned target pairing ‘rules’. In general, these algorithms produced lists of hundreds or even thousands of targets for a typical miRNA, of which a very limited number have ever been experimentally validated.

The majority of experimental ‘validations’ of computationally predicted miRNA:mRNA target pairs are performed in cell culture (e.g. luciferase reporter assays) or utilize transgenic over-expression assays, which can indicate the potential for regulation, but do not necessarily indicate that they are biologically relevant interactions. There is a growing body of literature which adds another layer of complication, suggesting that many miRNAs and their predicted mRNA targets are not expressed in the same cells. Instead, they are often expressed in complementary patterns, making in vivo validation of endogenous interactions very difficult [10].

Microarray data, both for miRNAs and putative mRNA targets, have been used to support the validity of the claims of hundreds of targets per miRNA [10]–[13]. This has proven to be effective to exclude genes as putative targets, as mRNAs that are co-expressed in a particular tissue with a given miRNA are found to be devoid of predicted target sites for that miRNA [10], [14]–[16]. Unfortunately, when mRNAs levels are altered in miRNA mutant animals or animals with ectopic overexpression of a given miRNA, this does not explicitly mean the mRNAs are directly targeted by the given miRNA. Additionally, quantitative mass spectrometry analysis of protein levels shows that a high proportion of predicted targets, even those which are conserved, are unaffected by changes in miRNA expression [17].

Recent studies have confounded matters even further. Systematic deletions of every miRNA gene in the *C.elegans* genome resulted in the surprising discovery that the vast majority had no significant phenotypes [18], [19]. Experiments using CLIP-seq/HITS-CLIP techniques have suggested that a large proportion of predicted miRNA-target sites are not bound by Argonaute proteins [20], [21].

The general lack of functional data and dearth of knowledge about concurrent expression of specific miRNAs and their putative targets at a cellular level in developing animals, have led to a situation where most of the proposed miRNA:mRNA target interactions found in databases are conjectural. Many of the aforementioned studies revealed that target pairing ‘rules’ are more complicated than previously assumed and that different miRNAs may have different sets of target pairing ‘rules’. Additionally, effective downregulation of a target genes may require additional context dependent sequences external to the target sites [22]–[25]. Taken together, these data imply that computational predictions may only be a reliable indicator of the target landscape for a limited number of miRNAs.

Despite the ambiguity involved in computational predictions, there are indications that Hox genes are likely to be an important class of miRNA targets. Evidence for post-transcriptional regulation of Hox gene expression has been accumulating for some time. Discrepancies have been found between transcript and protein levels for the mouse Hox gene *Hoxb4* in the posterior neural tube [26], *Hoxc6* in the chick hindlimb [27], and the the *Sex combs reduced* (*Scr*) ortholog in the first thoracic segment of *Porcellio scaber* [28]. It is currently unknown whether these discrepancies are due to miRNA regulation or other mechanisms such as localized protein instability.

In contrast to the modest number of documented cases of post-transcriptional Hox regulation, most Hox genes have been predicted to be direct targets of miRNAs in both vertebrates [29] and invertebrates [30]. One example of a Hox miRNA target being verified is the case of *Hoxb8* regulation by miR-196, which was shown in mice to cause endonucleolytic cleavage of the mRNA target site [31], [32]. This target site is conserved in vertebrate *Hoxb8* genes, and suggests that miR-196 has a conserved role in vertebrate axial patterning [33]–[35]. On the basis of partial complementarity between miRNA sequences and 3′ untranslated regions (UTR) sequences, Hox transcripts from the *Drosophila melanogaster Scr*, *Antp*, *Ubx*, *abd-A* and *Abd-B* genes have been proposed as targets of miRNA regulation [30], and several studies have been published in the last few years testing a number of these interactions in *Drosophila* [36]–[39], as well as analogous interactions in other animals [34], [40], [41].

The animal Hox complexes seem to be relatively rich in miRNAs, and considering the theory that miRNAs are spawned by neighboring genes which they can then go on to regulate [42], it seems compelling that these Hox cluster miRNAs might regulate nearby Hox genes, which they are often predicted to target. Most vertebrate and arthropod Hox complexes have at least three regions containing miRNA-producing hairpins. Although they reside in comparable positions in their respective Hox complexes, the arthropod miRNAs miR-iab-4 and miR-993 do not exhibit sequence homology to the vertebrate miR-196 and miR-615. On the other hand, miR-10 is a highly conserved miRNA, not only in sequence, but also in its genomic position in the complex between the *Hox4* and *Hox5* orthologs of most bilaterian animals [43].

In this study we investigated three strongly predicted miRNA:Hox-target interactions: miR-10-5p:*Scr*, miR-10-3p:*Abd-B*, and miR-iab-4-5p:*Antp*. Using various techniques we show that these predicted interactions do not appear to play detectable roles during embryonic development, and at least considered in isolation, have very subtle effects on Hox protein levels.

## Results

### Expression of *Drosophila pri-mir-10* transcripts

**Figure 1:**
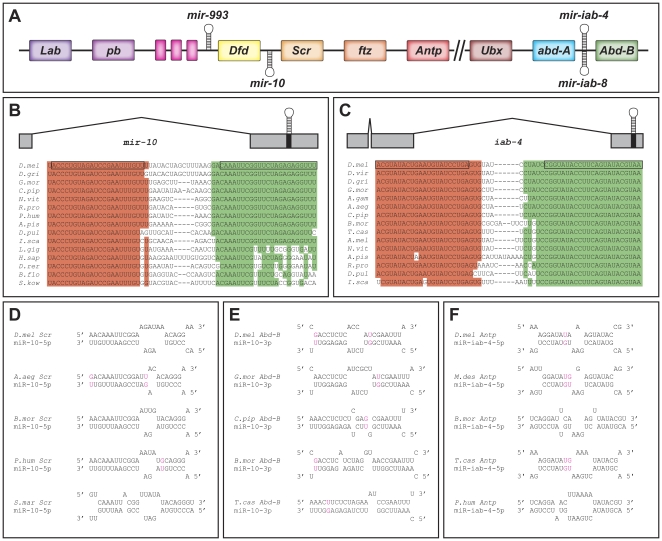
Hox complex miRNAs and their targets. (**A**) Cartoon of *Drosophila* Hox complex showing locations of miRNA hairpins. Unlabeled genes (pink boxes) are the 3 diverged Hox3 orthologs in *Drosophila zen*, *zen2*, and *bicoid*. // indicates a break in the Hox complex in *Drosophila melanogaster*. Transcription is generally from right to left. (**B**) Schematic of *mir-10* primary transcript and alignment of hairpin sequences from selected bilaterians. Boxed regions indicate miR-10-5p (red) and miR-10-3p (green) sequences. Shading indicates sequences highly conserved with *Drosophila*. (**C**) Schematic of *mir-iab-4* primary transcript and alignment of hairpin sequences from selected arthropods. Boxed regions indicate miR-iab-4-5p (red) and miR-iab-4-3p (green) sequences. Shading indicates sequences highly conserved with *Drosophila*. (**D**) Putative duplexes between miR-10-5p and 3′UTR sequences of *Scr* orthologs in selected arthropods. (**E**) Putative duplexes between miR-10-3p and 3′UTR sequences of *Abd-B* orthologs in selected insects. (**F**) Putative duplexes between miR-iab-4-5p and 3′UTR sequences of *Antp* orthologs in selected insects. The species shown in (D–F) were chosen to exhibit the ranges of miRNA:target site structures. More potential target sites for these miRNAs in different species are shown aligned in Figures S5, S6, and S9. Colored nucleotides in (D–F) indicate G:U base parings. *A.aeg* - *Aedes aegypti*, *A.gam* - *Anopheles gambiae*, *A.mel* - *Apis mellifera*, *A.pis* - *Acyrthosiphum pisum*, *B.flo* - *Branchiostoma floridae*, *B.mor* - *Bombyx mori*, *C.pip* - *Culex pipiens quinquefasciatus*, *D.gri* - *Drosophila grimshawi*, *D.mel* - *Drosophila melanogaster*, *D.pul* - *Daphnia pulex*, *D.rer* - *Danio rerio*, *D.vir* - *Drosophila virilus*, *H.sap* - *Homo sapiens*, *I.sca* - *Ixodes scapularis, L.gig* - *Lottia gigantea*, *L.lon* - *Lutzomyia longipalpis*, *M.dom* - *Monodelphis domestica*, *N.vit* - *Nasonia vitripennis*, *P.hum* - *Pediculus Humanus*, *R.pro* - *Rhodnius prolixus*, *S.kow* - *Saccoglossus kowalevskii*, *T.cas* - *Tribolium castaneum*.

miRNAs are initially transcribed as RNA polymerase II primary transcripts which are serially processed to produce mature miRNAs [44]. In order to determine the primary transcript that produces miR-10 (*pri-mir-10*), 5′ and 3′ RACE (rapid amplification of cDNA ends) were performed on a cDNA library created from polyadenylated (polyA) RNA from a 0–24 hour embryo collection. The 3′ RACE products reveal the polyA cleavage site to be about 700 bp downstream of the hairpin sequence (Figure 1A). 5′ RACE gave multiple products, the longest of which revealed exon-intron boundaries and a putative transcriptional start site about 6.8 kb upstream of the hairpin (Figure 1A). The sequence just upstream of the 5′ end of the RACE products contains properly spaced INR, DPE, and MTE elements [45], and is also well conserved amongst *Drosophilids* (Figure S1), which point to this region as a likely *pri-mir-10* promoter. We therefore conclude that at least one *pri-mir-10* transcript in *Drosophila melanogaster* spans an approximately 7.5 kb region of the chromosome between *Scr* and *Dfd* with a single approximately 5.5 kb intron (Figure 1A and B), although the existence of secondary transcripts remains a possibility.

**Figure 2:**
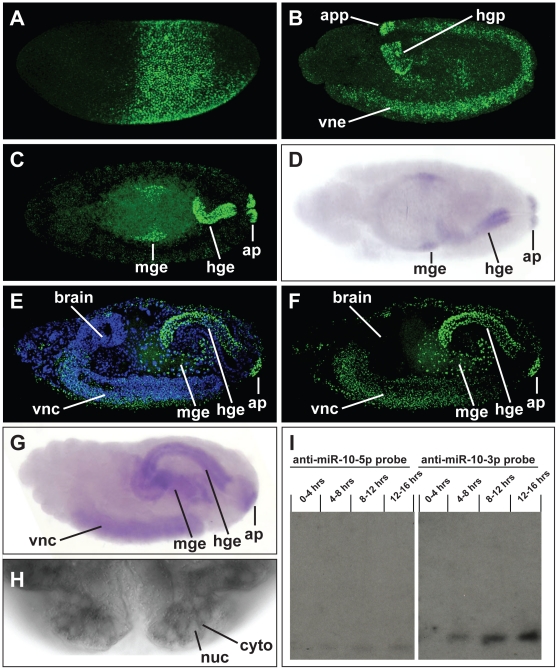
*mir-10* expression during embryogenesis. (**A**) *pri-mir-10* is expressed in an early gap-gene like pattern in blastoderm stage embryos. (**B**) In germband extended embryos *pri-mir-10* transcription begins in the anal pad primordia (app) and hindgut primordia (hgp) as well as ventral neurectoderm (vne). (**C**) In stage 14 embryos *pri-mir-10* staining is seen in the developing anal pads (ap), hindgut endoderm (hge) of the large intestine, a small patch of midgut endoderm (mge), and continues to be expressed in the developing nerve cord (out of view). (**E,F**) In late stage embryos the domains of expression are maintained and CNS staining has an anterior boundary near the sub-esophageal ganglion and extends to near the posterior end of the ventral nerve cord. Starting around stage 13 both mir-10-5p (**D**) and miR-10-3p (**G**) mature miRNAs are detected by LNA in situ in the same patterns as for *pri-mir-10* (compare to C and F respectively). (**H**) Detection of miR-10-3p shows mature miRNA is localized in the cytoplasm. (**I**) Northern blots of total RNA purified from *Drosophila* embryos at various developmental stages using ^32^P end labeled antisense oligo probes against miR-10-5p and miR-10-3p. Time course shows both miR-10-5p and miR-10-3p are produced at very low levels in blastoderm embryos and during early germ band extension (0–4 hrs) and progressively increase in abundance throughout embryogenesis. miR-10-3p is found at much higher levels throughout embryogenesis when compared to miR-10-5p.

In order to determine the expression pattern of the *mir-10* gene during embryogenesis, we performed in situ hybridizations using probes antisense to *pri-mir-10* sequence and analyzed expression throughout embryogenesis. *mir-10* primary transcripts are first expressed during the blastoderm stage of embryogenesis [46]. At this point *pri-mir-10* is expressed in a broad band which corresponds to the trunk primordia of the embryo and is reminiscent of gap gene or Hox gene expression patterns (Figure 2A). The expression pattern at this stage is largely complementary to that of *hunchback*, suggestive of a possible negative regulatory interaction. However, expression of *pri-mir-10* was unaltered when examined in embryos mutant for the *hunchback* gene (data not shown). The *mir-10* primary transcript was also detected in yolk cells of blastoderm stage embryos (data not shown). During late blastoderm development and the beginning of gastrulation the expression of *pri-mir-10* becomes downregulated in a subset of cells, taking on a striped appearance, which is similar to, but not as refined as that of the pair rule genes (data not shown). These stripes are approximately in register with those of the Hox complex gene *fushi tarazu* (data not shown). Shortly after the beginning of gastrulation, transcription of *pri-mir-10* appears to shut off and then re-initiates during the early stages of germband elongation in a different pattern. As germband elongation proceeds, *pri-mir-10* transcription is initiated in the anal pad and large intestine primordia and in ventral neurectoderm of the trunk segments where it is expressed in the developing neuroblasts (Figure 2B). After germband retraction *pri-mir-10* is transcribed in the anal pads and large intestine endoderm as well as a subsection of the central midgut endoderm (Figure 2C). In late embryogenesis *pri-mir-10* staining can be seen in the anal pads, large intestine endoderm, midgut endoderm, and in the ventral nerve cord of the central nervous system (CNS) (Figure 2E–F). The anteroposterior extent of *pri-mir-10* expression in the CNS is reminiscent of its blastoderm expression pattern. No *pri-mir-10* transcription is detected in the brain, with the anterior border of expression in the posterior region of the sub-esophageal ganglion, extending posteriorly to near the terminal end of the CNS (Figure 2E–F). It is possible that this pattern of expression in the CNS may be conserved in all bilaterians as a similar expression pattern is seen in zebrafish embryos [47], [48].

### Expression of mature miRNAs from *pri-mir-10*

After a pre-miRNA is cleaved by Dicer, one strand of the resulting ∼22 nt dsRNA is packaged into an RNA Induced Silencing Complex (RISC). The strand with a lower 5′ binding energy is typically packaged more often into RISC as a mature miRNA [49]. This type of analysis predicts that there is no biochemical reason why *Drosophila* miR-10-5p (previously called miR-10) should be preferentially packaged into RISC more often than miR-10-3p (previously called miR-10*), and in fact suggests that miR-10-3p should be packaged into RISC preferentially over miR-10-5p ([49] and data not shown).

Both miR-10-5p [50], [51] and miR-10-3p [50] were cloned in early efforts to determine the miRNA complement of the *Drosophila* genome. However, the data from these studies was not enough to statistically determine the relative abundance of either strand for most miRNA duplexes. Due to the presence of miR-10-5p sequences in most other animals the possibility of a second miRNA from this locus was largely ignored. Although the sequence from the 5′ arm of the *mir-10* hairpin is well conserved in bilaterians, the sequence from the 3′ arm is not particularly well conserved. The conservation that does exist among disparate animal clades (Figure S2) may be due mostly to the necessity for complementary bases which allow formation of a hairpin capable of being processed by the miRNA machinery. However, while the 3′ hairpin sequence is not well conserved in deuterostomes (Figure S3B), if only arthropods are examined, both miR-10-5p and miR-10-3p sequences are found to be well conserved (Figure S3A). Additionally, although the putative seed sequence of the potential deuterostome miR-10-3p is conserved, free energy calculations suggest that it is unlikely to be packaged into RISC at appreciable levels (data not shown).

In order to test the abundance and timing of expression of the products of the *mir-10* duplex, Northern blot analysis was performed on RNA from multiple embryonic stages using antisense probes complementary to each arm of the hairpin. Both miR-10-5p and miR-10-3p were found to be present in embryos, with levels increasing throughout embryogenesis, and with miR-10-3p present at significantly higher levels at all stages (Figure 2I). These data agree well with recent large scale miRNA sequencing efforts which profile expression levels of all mature products of a miRNA gene [52]. In addition to Northern analysis of miRNAs, mature miRNAs can be detected by in situ hybridization by using modified oligonucleotides composed of Locked Nucleic Acid (LNA) nucleotides [47]. Both miR-10-5p (Figure 2D) and miR-10-3p (Figure 2G) can be detected in late embryonic stages utilizing this technique, and the expression pattern of each is equivalent to *pri-mir-10* (compare Figure 2D to 2C and Figure 2G to 2E). Additionally, staining for the mature miRNAs is in the cytoplasm (Figure 2H), unlike primary transcript staining, which is found exclusively in the nucleus.

### Identification of putative targets

Due to the conserved location of the *mir-10* gene in animal Hox complexes (Figure S4), and the potential for miRNAs to be spawned from, as well as to target nearby genes [42], we were interested in the possibility that miR-10-5p and miR-10-3p might be regulators of Hox gene expression. Simple sequence motif searches of the 3′UTR sequences of *Drosophila* Hox genes resulted in identification of potential target sites of varying strength.

The best putative target site for miR-10-5p was found in the 3′UTR of the Hox gene *Sex combs reduced* (*Scr*). Although the 5′ seed sequence pairing of miR-10-5p with this *Scr* site is not as extensive as the ‘prototypical’ mRNA pairing—a continuous helix of base pairs 3–7, as opposed to the ‘ideal’ seed sequence of miRNA nucleotides 2–8—there is potential for a long continuous helix formed between *Scr* and the 3′ end of the miR-10-5p (bases 11–22) (Figure 1D and Figure S5), suggesting formation of an energetically stable duplex with miR-10-5p primed RISC. While not a top hit in most computational predictions due to the shortened seed match, *Scr* has been previously suggested as a target for miR-10-5p [30]. Analysis of the putative *Scr* 3′UTR sequences from other insects indicates that the predicted interacting bases are extremely well conserved in nearly all species examined, despite the fact that the neighboring sequences have diverged (Figure S5), suggesting specific selection since the evolutionary split between the major insect groups more than 300 million years ago. In fact, the putative target sites from most of the non-*Drosophilid* insects display even higher complementarity to the seed region, which could provide added strength to the potential interaction. We also used the RNAHybrid program [53], and our analysis identified this *Scr* sequence as one of the strongest putative targets for miR-10-5p within all *Drosophila* 3′UTRs (data not shown). Although miRNA target prediction algorithms have had varying success at prediction of true positive miRNA-mRNA interactions [54], its status as the top hit in our predictions, along with the conservation in arthropods, suggests that *Scr* is one of the best putative targets of miR-10-5p in *Drosophila*.

We also identified a putative target site for miR-10-3p in the 3′UTR of the Hox gene *Abdominal-B* (*Abd-B*) (Figure 1E). Similar to the putative target site for miR-10-5p in the *Scr* 3′UTR, this sequence appears as one of our top hits among all *Drosophila* 3′UTRs using RNAHybrid, contains potential long continuous helix pairings with the miRNA, and is well conserved in the putative 3′UTR sequences of most insect *Abd-B* orthologs (Figure 1E and data not shown). This site is also conserved within a conserved block sequence surrounded by non-conserved 3′ UTR sequence among fly *Abd-B* orthologs (Figure S6). Additionally, among our top predicted miR-10-3p targets, this is the only site with significant evolutionary conservation (data not shown). This target site for miR-10-3p in the *Abd-B* mRNA was also predicted in a previous study [55].

### Functional analysis of miR-10-5p

miRNAs may function in a number of ways to downregulate their target genes. Most initial studies indicated that miRNA targeted genes were downregulated by inhibition of protein production without affecting the levels of cytoplasmic mRNA [56], suggesting one could look for discrepancies between mRNA and protein levels for a given gene in order to identify regions of potential miRNA regulation. While there have been no published examples of post-transcriptional regulation of *Scr* during *Drosophila* embryogenesis, there is apparent post-transcriptional regulation of the *Porcelio scaber Scr* ortholog in the maxilliped segment during embryogenesis [28]. This led us to reexamine the expression pattern of *Scr* transcripts and protein more closely.

The expression of mRNA and protein products from the *Scr* gene overlap, for the most part, in all tissues during embryogenesis. However, in germband extended embryos, there is field of cells in the ventral first thoracic segment which transcribes *Scr* and contains low levels of *Scr* cytoplasmic mRNA, but that never exhibits any detectable SCR protein accumulation (Figure S7). During this stage, *pri-miR-10* is not actively transcribed in all of these cells, but it seemed possible that miR-10-5p produced from early transcripts might still be present in these cells to inhibit translation. However, LNA antisense oligo staining did not detect any mature miR-10-5p in these cells at this stage (data not shown). Furthermore, in situ analysis using a probe flanking the 3′ of the miR-10 hairpin region suggests that *pri-miR-10* transcripts that include a polyadenylation signal are not made in the ectoderm of early blastoderm embryos (data not shown). Taken together these data suggest that, although *Scr* appears to be post-transcriptionally regulated in these cells, it is not through the activity of miR-10-5p.

No other regions exhibiting discrepancies between *Scr* transcripts and protein were found during *Drosophila* embryogenesis. The other possibility was that miR-10-5p might be acting through *Scr* mRNA degradation as has been shown for a number of miRNA targets [57], [58]. If this were the case, then transcript pattern and/or levels would be altered in embryos ectopically expressing or lacking the *mir-10* gene. Unfortunately, available chromosomal deletions which lack *mir-10* sequences also delete *Scr*, precluding the possibility of ascertaining the change in *Scr* expression in these mutant embryos.

In order to test whether *Scr* expression is altered in embryos that ectopically express *mir-10*, transgenic flies were constructed that allow expression of *pri-mir-10* under the control of a variety of spatially and temporally specific GAL4 drivers. The *pri-mir-10* transgene can produce significant amounts of mature miRNA, as visualized by antisense LNA oligo staining in embryos containing both the UAS-*mir-10* transgene as well as a *prd*-GAL4 driver (Figure S8A).

Ectopic expression of *pri-mir-10* by GAL4 drivers that persistently produce *pri-mir-10* in all embryonic cells results in death during early larval development and minor cuticle phenotypes in the anterior of the early larvae. The transgenic larvae die typically during the first instar and occasionally do not hatch, but none survive to adulthood. These larvae have malformations of their anterior cuticle which appear as cuticular breaks near the mouthhooks (compare Figure S8C–D with Figure S8B). Although these cuticle phenotypes are reproducible, they do not obviously correlate with known embryonic loss of function *Scr* mutant phenotypes, which include reduction of the first thoracic beard structure, and labial sense organ abnormalities [59], [60].

**Figure 3:**
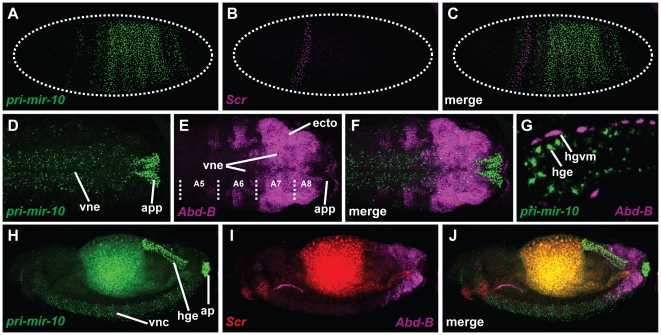
*pri-mir-10* is expressed in a pattern complementary to predicted targets. Images of in situ hybridizations of *pri-mir-10*, *Scr*, and *Abd-B* at various stages of development. (**A**) *pri-mir-10* and (**B**) *Scr* in a blastoderm stage embryo. (**C**) Overlay of (A) and (B). (**D**) *pri-mir-10* and (**E**) *Abd-B* expression in the posterior of the extended germband of a stage 11 embryo. (**F**) Overlay of (D) and (E). (**G**) Field of cells from the large intestine of the hindgut of a stage 15 embryo. *pri-mir-10* is expressed in the endodermal cells, while *Abd-B* is expressed in the visceral mesoderm surrounding the gut. (**H**) *pri-mir-10* and (**I**) *Scr* and *Abd-B* staining in a stage 15 embryo. (**J**) Overlay of (H) and (I). Note overlapping expression in posterior neurectoderm.

**Figure 4:**
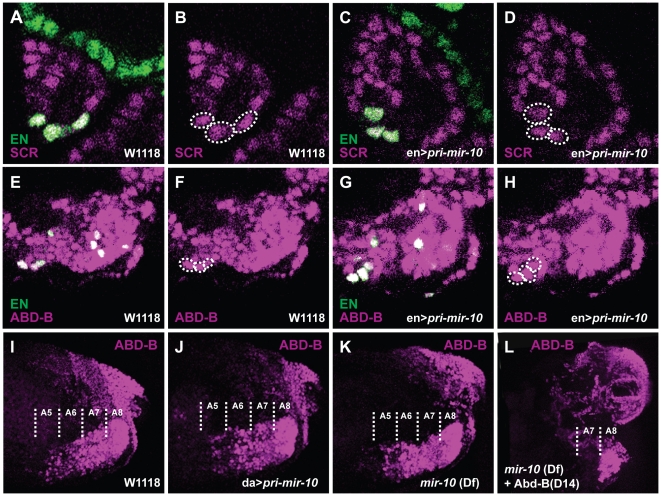
Overexpression or lack of *pri-miR-10* has no observable effect on putative target protein expression. Embryos expressing UAS-*pri-miR-10* under the control of *engrailed-*GAL4 (en>*pri-miR-10*) (**C,D,G,H**) exhibit no consistent detectable decrease in either SCR protein (**A–D**) or ABD-B protein (**E–H**) in cells expressing ectopic miRNA [marked with anti-*en* antibodies (green), or circled with dotted lines] versus control cells in wild type embryos (**A,B,E,F**). No consistent detectable change in ABD-B protein levels is seen in the CNS of embryos ubiquitously expressing UAS-*pri-miR-10* under the control of *da*-GAL4 (da>*pri-miR-10*) (**J**) or in embryos deficient for the *mir-10* locus ((*Df3R*)*CP1*) (**K**) compared to wild type embryos (**I**). Embryos mutant for the *Abd-B*-m specific transcripts (*Abd-B^D14^*) and deficient for *mir-10* do not exhibit ectopic r-type ABD-B protein (**L**).

*Scr* and *pri-mir-10* are expressed in largely complementary patterns in both early embryonic ectoderm (Figure 3A–C) and in late stage CNS (Figure 3H–J). This correlation is consistent with the hypothesis that miR-10-5p is negatively regulating *Scr* mRNA levels, but ectopic expression of *pri-mir-10* did not provide any support for this hypothesis. The *engrailed*-GAL4 driver expresses in a small number of cells which overlap the *Scr* expression pattern. When *pri-mir-10* is expressed under the control of *engrailed-*GAL4, these cells have no obvious decrease in SCR protein levels when compared to wildtype controls (compare Figure 4C–D with Figure 4A–B). Additionally, embryos ectopically expressing *pri-mir-10* from a variety of other GAL4 drivers did not exhibit detectable changes in either pattern or levels of *Scr* transcript or protein accumulation (data not shown).

### Functional analysis of miR-10-3p

In order to characterize a possible interaction between miR-10-3p and *Abd-B,* we used in situ hybridization to determine their relative patterns of expression. During embryogenesis *pri-miR-10* and *Abd-B* are expressed in mostly non-overlapping regions. While *Abd-B* is expressed in the majority of the posterior ectoderm, it is completely excluded from the developing anal pads, where *pri-miR-10* and miR-10-3p are strongly expressed (Figure 2G–H; Figure 3D–F and H–J). *Abd-B* is also expressed throughout later embryogenesis in the visceral mesoderm surrounding the large intestine of the hindgut, whereas miR-10-3p is expressed only in the endodermal cells of this region (Figure 3G). This complementarity is suggestive of either unidirectional or mutual negative regulatory interactions. However, embryos which are mutant for *Abd-B* gene products do not show altered expression of *pri-mir-10* (data not shown). Conversely, embryos which are deleted for the *mir-10* gene (*Df(3R)CP1*), and thus lacking miR-10-3p [46], do not exhibit ectopic expression of *Abd-B* mRNA or protein in either the large intestine endoderm or in the anal pads, suggesting that miR-10-3p does not regulate *Abd-B* in these tissues (data not shown).

Although their transcription is largely complementary in most tissues, in the ventral nerve cord of later stage embryos, *pri-mir-10* and *Abd-B* overlap from the posterior compartment of segment A5 through A8/9 (parasegments 10–14) (Figure 3D–F and H–J). The *Abd-B* gene produces multiple transcript variants which code for two different proteins. Of these, the shorter ABD-B r-type protein is produced in the CNS only in the most posterior regions (A8/9), whereas the longer m-type protein is expressed in more anterior regions of the CNS (A5–A8) [61]. Additionally, *Abd-B* may utilize either of two polyA signals to terminate transcripts [62]. The longer *Abd-B* 3′UTR contains the putative miR-10-3p target site, while the shorter 3′UTR lacks the target site. Most tissues only produce *Abd-B* transcripts with the shorter 3′UTR, while the longer 3′UTR is only found on *Abd-B* transcripts in the CNS ([63], and data not shown). Although it is not known whether the longer UTR belongs exclusively to transcripts that encode either ABD-B m-type or r-type proteins, the longer UTR has been found on transcripts that encode ABD-B-r [62]. Since the domain of *pri-mir-10* transcription extends into the posterior of the CNS, but appears to decrease toward the extreme posterior (Figure 3H, J), we hypothesized that miR-10-3p might be repressing r-type ABD-B protein in more anterior segments of the CNS. To test this possibility, *Abd-B* ^D14^ (an mutant that eliminates only ABD-B m-type protein) was recombined with *Df(3R)CP1*. This strain produces embryos which are deficient for both miR-10-3p and ABD-B m-type protein. Analysis of ABD-B protein staining does not show any anterior expansion of ABD-B r-type protein expression in this mutant background (Figure 4L). This suggests that the longer 3′UTR staining detected in A5–A7 are found on transcripts encoding ABD-B m-type protein.

To test the possibility that miR-10-3p is regulating *Abd-B* expression in the CNS more generally, transheterozygote *Df(3R)CP1*: *Df(3R)LIN* embryos deficient for *mir-10* were analyzed for alterations in *Abd-B* transcript and protein levels in segments A5–A8/9 of the CNS. No consistent differences were seen in the expression levels or anterior extent of expression of either *Abd-B* transcripts (data not shown) or protein (Figure 4K) when compared to wildtype embryos (Figure 4I), although in A5–6, occasional mutant embryos showed slightly higher levels of ABD-B protein staining. Ectopic expression of *pri-mir-10* in the CNS either through ubiquitous overexpression (Figure 4J) or in a subset of cells (Figure 4G, H) did not result in obviously reduced levels of ABD-B protein compared to wildtype (Compare to Figure 4E, F, and I).

### bithorax complex miRNAs and ANTP regulation

In *Drosophila*, the bithorax complex (BX-C) is a large genomic region that is responsible for patterning much of the abdominal body region [64]. Although early genetic studies of the BX-C found that it contained only the protein-coding genes *Ubx*, *adb-A*, *and Abd-B* [65], [66], large portions of the intergenic regions are also transcribed, giving rise to numerous long non-coding RNAs [67]. While the exact function of these RNAs is unclear, it is believed that early transcription through these regions is necessary to activate cryptic Hox enhancers, and primes the complex for later epigenetic regulation [68]–[70].

**Figure 5:**
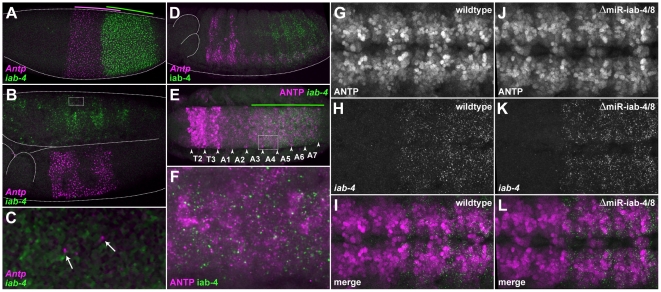
*iab-4* is coexpressed with *Antp* in a subset of embryonic cells, but does not appear to play a large role in repressing translation of endogenous transcripts. (**A–D**) Fluorescent in situ hybridization was carried out using probes specific for *Antp* transcripts and for the long intron of *iab-4*, which are shown in magenta and green, respectively. (**A**) A blastoderm-stage embryo. Colored lines indicate the extents of the gap-gene-like expression patterns for each gene. (**B**) The trunk of a stage-11 embryo showing *Antp* and *iab-4* expression. (**C**) A close-up view of the dashed box indicated in (B) shows apparently spurious *Antp* expression (arrows) in the abdominal segments that occasionally appear in cells that express *iab-4*. (**D**) In a stage-14 embryo, *Antp* expression is found mainly in the ectoderm of the second and third thoracic segments, and in segments T2–A7 of the ventral nerve cord (VNC); *iab-4* expression is found mainly in the lateral ectoderm and in the VNC of abdominal segments A4–A7. (**E**) ANTP protein (magenta) and *iab-4* transcripts (green) are coexpressed in neuromeres A3–A7 in the developing VNC of a stage-14 embryo. The extent of *iab-4* expression is indicated with a green line. (**F**) A close-up view of the dashed box indicated in (E) displaying the largely complementary nature of ANTP and *iab-4* expression in the abdominal VNC. (**G–L**) ANTP expression appears largely unchanged in embryos homozygous for a *miR-iab-4/8* hairpin deletion when compared to heterozygous embryos. (**G**) ANTP expression in neuromeres A1–A4 of a stage-14 wildtype embryo (Δ*miR-iab4/8/TM3,Ubx-lacZ*). (**H**) *iab-4* expression in the same region as (G). (**I**) A merged image of (G) and (H). (**J**) ANTP expression in neuromeres A1–A4 of a stage-14 homozygous miRNA-deficient embryo (Δ*miR-iab-4/8*). (**K**) *iab-4* expression in the same region as (J). (**L**) A merged image of (J) and (K).

It is now also clear that at least 2 of these long non-coding RNAs, *iab-4* and *iab-8*, also contain hairpin precursors for three miRNAs: miR-iab-4-5p, miR-iab4-3p, and mir-iab-8-5p (also referred to as miR-iab4AS-5p) [37]–[39]. Interestingly, primary transcripts that encode miR-iab-4 are expressed principally in A4–7, with lower levels produced in A2–3 ([36]–[39], and Figure 5 A–E). Transcription of *iab-8* produces miR-iab-8-5p in abdominal segments 8–9 from the opposite strand of the same hairpin [37]–[39]. The hairpin-encoding sequence that generates these miRNAs can be found in the genomes of all sequenced arthropods in a conserved position between the *abd-A* and *Abd-B* orthologs (Figure 1A). Except for a few minor changes, the miR-iab-4/miR-iab-8 encoding sequences of both arms of the hairpin are completely conserved (Figure 1C).

These miRNAs have been shown several times to be present throughout embryogenesis via cloning and Northern analyses [37]–[39], and have all been predicted to regulate one or more of the Hox genes. A number of recent studies have presented evidence supporting several of these predicted interactions: miR-iab-4-5p:*Ubx* [36], [39], miR-iab-8-5p:*Ubx* [37]–[39], and miR-iab-8-5p:*abd-A* [38], [39]. The best genetic evidence for a biologically relevant functional interaction is for miR-iab-8-5p as a negative regulator of UBX protein levels in a subset of CNS cells in the 8th abdominal segment [37].

In addition to these interactions, it has also been predicted that miR-iab-4-5p or miR-iab-8-5p might also bind to the 3′UTR of the Hox gene *Antp* [38], [39]. Through our own analysis we identified a strong putative binding site for miR-iab-4-5p in the 3′UTR of *Antp*. This site was not found in previous predictions due to its lack of canonical seed pairing. However, this site does have prototypical miRNA/mRNA 5′ and 3′ helical pairing, and similar putative target sequences can also be found downstream of *Antp* orthologs from many other insects (Figure 1F). Although the conservation of this sequence is not as strong as the predicted miR-10-5p target in *Scr* orthologs (Figure S5), the *Antp* sequences that would pair with miR-iab-4-5p are completely conserved amongst *Drosophila* species (Figure S9).

### Functional analysis of miR-iab-4-5p and *Antp*

To investigate a possible interaction between *Antp* and miR-iab-4-5p, we performed in situ hybridizations on *Drosophila* embryos with probes against the *pri-mir-iab-4* (*iab-4*) and *Antp* transcripts (Figure 1C, and Figure 5A–D). During early embryogenesis *Antp* and *iab-4* are expressed in broad, partially overlapping patterns throughout the future trunk and abdomen (Figure 5A). However, it is unlikely that co-expression at this stage is informative, as the *Antp* transcription unit is quite long and does not have time to produce full-length mRNAs during the short nuclear division cycles of the blastoderm [71]. During germband extension and retraction these patterns refine, with the major domain of co-expression corresponding to the CNS of abdominal segments 3 through 7 (Figure 5B–D). It is also interesting to note that the transcription pattern of *Antp* seems ‘noisier’ than that of other Hox genes, as low numbers of transcripts are often observed in the ectoderm of the abdomen in variable patterns (Figure 5C, arrows). As these never seem to correlate with detectable ANTP protein levels (data not shown), it is possible that one of the functions of the *iab-4* miRNAs might be to repress translation of these apparently spurious *Antp* mRNAs. Unfortunately, the rare and stochastic nature of these transcripts does not allow any firm conclusions to be drawn concerning a role for miR-iab-4-5p in the suppression of ectopic protein production.

The main region of co-expression between ANTP protein and *iab-4* is in the CNS of abdominal segments 3–7 of later stage embryos (Figure 5E–F). Looking closely at the expression patterns, we see that in regions of overlapping expression, most cells with high levels of *iab-4* expression have low levels of ANTP protein, which is at least consistent with potential miR-iab-4 regulation of ANTP levels (Figure 5E–F).

To determine whether significant regulation occurs in vivo in the region of overlapping expression, we took advantage of a *Drosophila* strain in which the *mir-iab-4/8* hairpin has been precisely deleted [37]. Conveniently, as the rest of the primary transcript is still produced as normal in these mutants, we can still stain for the *iab-4* precursor RNA to pinpoint areas that might now contain higher ANTP levels. However, no obvious reproducible changes in ANTP protein levels were seen in the co-expressed regions throughout embryogenesis (compare Figure 5G–I to Figure 5J–L). Occasionally, *mir-iab-4/8* embryos would be observed in which certain subsets of neuroblasts displayed higher ANTP levels in areas of *iab-4* co-expression (data not shown). However, the rarity of these events prevented rigorous quantification of this phenomenon.

Finally, to determine whether the function of miR-iab-4-5p was to buffer ANTP levels against environmental fluctuations [72]–[74], we subjected developing wildtype and *miR-iab-4/8* mutant embryos to several rounds of temperature shifts (see methods). Again, we saw no reproducible changes in ANTP protein levels in *miR-iab-4/8* mutants versus wild type, even under these stressful developmental conditions (data not shown).

## Discussion

A growing body of circumstantial evidence and computational predictions have consistently pointed to *Drosophila* Hox genes as a significant class of miRNA targets. Our results do not support the idea that *Drosophila* Hox complex miRNAs play a significant embryonic patterning role through the regulation of their predicted targets in Hox protein-coding messenger RNAs. This is consistent with previous loss-of-function experiments on the role of the *mir-iab-4/8* genes, which indicated that regulation of a Hox target gene (*Ubx*) by miR-iab-8-5p led to only subtle changes in the UBX protein expression pattern in embryos, and no detectable changes in axial body patterning [37]. In *C. elegans*, the study of *mir-57*, which is likely to be a derived ortholog of *mir-10*, indicates that loss-of-function mutants have partially penetrant posterior patterning defects that may in part be due to the derepression of *nob-1b*, a *C. elegans* ortholog of *Abd-B* [41]. It is possible in vertebrates, that Hox complex miRNAs play a greater role in body patterning, as their functional knockdown using antagonistic complementary nucleic acids have led to dramatic changes in Hox protein-coding gene expression and body patterning defects [34], [35], [48]. If the predicted Hox miRNA:Hox RNA target interactions in *Drosophila* are functional in vivo, why are they so difficult to observe? Both the miRNAs and their target sites have been conserved over vast evolutionary time in insects, so they must be under strong selective pressure.

Based on the current evidence, we believe that the evolutionarily conserved function of Hox miRNAs in bilaterians is most likely to subtly modulate Hox protein levels in sub-regions of the CNS. In addition to a few examples in *Drosophila* based on loss-of-function miRNA mutants [37], [63], there is compelling evolutionary data to support this theory. In addition to being conserved in both sequence and genomic position, *mir-10* appears to have a conserved expression pattern in the CNS of *Drosophila* and zebrafish [47], [48]. Members of the vertebrate specific Hox miRNA family, *mir-196*, are also expressed in the CNS of zebrafish [35], [47] and chicken [75], and *mir-iab-4* and *mir-iab-8* (while not orthologous by sequence, but orthologous by chromosomal position in the Hox complex) also are expressed in the *Drosophila* CNS ([36]–[39], and Figure 5). Additionally *mir-993* is also expressed in the *Drosophila* CNS from around the subesophageal ganglion to the posterior abdominal segments (data not shown).

When considering miRNA regulation of Hox genes in the CNS, an added layer of complexity must also be considered. *Ubx*, *Antp*, *abd-A*, *and Abd-B* all appear to produce both short and long forms of their 3′UTRs in various sub-regions of their expression patterns [63]. This could result in a variable mixture of miRNA sensitive and insensitive Hox transcripts, which might be utilized by cells to modulate protein levels in the presence of static miRNA expression. Alternative UTR usage with and without miRNA target sites may be a mechanism utilized by many tissues and cells in general [16]. While very intriguing, this further complicates efforts to characterize endogenous miRNA:target interactions, as down-regulation may be masked by translation from miRNA insensitive transcripts.

Another scenario is that the major function of the Hox miRNAs, many of which have expression patterns that are complementary to their predicted target Hox mRNAs, function largely to suppress aberrant Hox protein expression outside of their normal functional domains. Most ‘validations’ of miRNA:Hox interactions have only demonstrated that misexpression of *Drosophila* Hox miRNAs in regions where they are not normally found can result in homeotic transformations [36], [38], [39]. Bender [37] showed that while the function of miR-iab-4-5p and -3p appear dispensable for normal development, only the loss of miR-iab-8-5p led to male sterility, possibly due to the expansion of UBX protein expression into some cells in the most posterior segments where it is not normally expressed. Evidence has indicated that many miRNAs and their most strongly predicted targets are often expressed in mutually exclusive patterns [10], which is consistent with what we observed for miR-10-5p:*Scr* and miR-10-3p:*Abd-B*, limiting the potential for regulation of Hox protein expression during embryonic development.

Although we were unable to detect evidence for canalizing activity of the Hox miRNAs we tested, our results and others are consistent with the hypothesis that one major role of miRNAs is to canalize expression of their target genes in order to achieve precise reproducible spatial limits and levels of expression in order to maintain a reproducible phenotypic output in spite of environmental fluctuations [72], [74]. This attractive, yet difficult to demonstrate hypothesis has some experimental support [41], [73]. Our own attempts at inducing aberrant ANTP protein accumulation in *iab-4/8* mutants by subjecting embryos to temperature fluctuations were unsuccessful, although recapitulating all the stresses encountered by a developing *Drosophila* embryo in the wild is difficult to exhaustively test in a laboratory context. Even if canalization was the sole function of many miRNAs, it could account for the observed conservation of both miRNAs and their target sites throughout evolution. It is also possible that in the lineage leading to Drosophila, Hox miRNA functions have become less important in early developmental patterning than in other insect lineages, although important enough to largely conserve their target site sequences in Hox protein coding mRNAs. miRNA genes have been residents of animal Hox clusters for more than 500 million years, and it is conceivable that the gain or loss of Hox miRNA target sites, or changes in spatio-temporal expression of Hox-targeting miRNAs could have played an important role in the evolution of Hox gene function during animal evolution.

## Materials and Methods

Cuticle preparations were performed as in [76]. Northern blots were performed as in [77]. Standard in situ hybridizations and immunohistochemistry were performed as in ([46]; http://biology.ucsd.edu/~davek), except when simultaneously detecting RNA and protein, acetone was substituted for Proteinase K for permeabilization, to preserve epitope integrity [78], [79]. LNA in situ hybridizations were performed as in [80], modified for *Drosophila* with embryo preparation as in [46], using 5′ DIG labeled miRCURY LNA™TM probes from Exiqon.

### Sequences and abbreviations

Sequences were obtained using BLAST searches of NCBI sequence databases (http://blast.ncbi.nlm.nih.gov/Blast.cgi). Sequence alignments were created using BioEdit software [81].

### Fly stocks and embryo collection

*Df(3R)CP1*; *Df(3R)LIN*; *ΔmiR-iab-4/8*/*TM3,Ubx-LacZ* flies were a gift from W. Bender [37]. Unless otherwise noted, all embryos shown are of the genotype *w^1118^* (Bloomington Stock Center, http://flystocks.bio.indiana.edu). Embryos were raised, collected, and fixed as reported elsewhere ([46]; http://biology.ucsd.edu/~davek).

### Antibodies

Mouse primary antibodies against Hox proteins were obtained as concentrates from the Developmental Studies Hybridoma Bank and used at 1∶300 dilutions: SCR (6H4.1), ABD-B (1A2E9), and ANTP (4C3). The EN antibody was a gift from Patrick O'Farrell (1∶300 dilution). The SCR antibody used in Figure S7 for simultaneous detection with in situ probes was a gift from Debbie Andrews (rabbit polyclonal anti-SCR; 1∶100 dilution) Primary antibodies against haptens were obtained from Roche, and used at 1∶800 dilutions: mouse anti-BIO; sheep anti-DIG; rabbit anti-DNP; guinea pig anti-FITC; Alkaline Phosphatase (AP) conjugated sheep anti-DIG; and Horseradish Peroxidase (HRP) conjugated sheep anti-DIG. Alexa Fluor labeled secondary antibodies were obtained from Molecular Probes/Life Technologies were used at 1∶400 dilutions: donkey anti-sheep Alexa647; donkey anti-mouse Alexa488; donkey anti-rabbit Alexa555; and goat anti-guinea-pig Alexa594.

### *In situ* probes

Unless otherwise noted, antisense RNA probes were created by cloning appropriate PCR fragments into the pCR II vector (Invitrogen, K207040), and hapten-tagged probes were transcribed in vitro, as described elsewhere [46]. Hapten tags included: biotin (BIO), digoxigenin (DIG), fluorescein (FITC), and dinitrophenyl (DNP). When probes are referred to as ‘unfragmented’, this means that chemically fragmented probes did not produce high-quality stains, so unfragmented probes were used. Primers used in cloning probe sequences: *mir-10* (Figure 2) (5′-GCATTTCTACCTGCCTCTCG-3′ and 5′-GCTTGCCATCAGCAACACT-3′); *Scr intron* (5′-CACTTCTGCGAGACACTTGC-3′ and 5′-CAACCCCAGTTCCCATACAG-3′); unfragmented; *iab-4-*DIG and *iab-4*-DNP (5′-ACCACAAGAAGGAGCAGTCG-3′ and 5′-GCACTCTCACCTACACGAATGC-3′); *pri-mir-10* probes (Figure 3) were made from the *pri-miR-10* cDNA (GeneBank); *Scr* mRNA probes were made from a full length *Scr* cDNA (gift from J. Mahaffey); *Abd-B* mRNA probes were transcribed from an *Abd-B* cDNA [62]; *Antp-*P1-BIO was made as described elsewhere [82]; *LacZ*-FITC was transcribed from the pBS-LacZ plasmid.

### Detection schemes

To detect *pri-mir-10* in Figure 2 the detection scheme was as follows: *mir-10*-DIG>sheep anti-DIG-HRP>Cy3 Tyramide amplification;

To detect *pri-mir-10*, *Scr*, and *Abd-B*, the detection scheme was as follows: *pri-mir-10*-DIG>sheep anti-DIG-HRP>Cy3 Tyramide amplification; *Scr*-cDNA-BIO>mouse anti-BIO>donkey anti-mouse Alexa488; *Abd-B*-cDNA-DNP>rabbit anti-DNP>donkey anti-rabbit Alexa647.

To detect *Scr* mRNA, *Scr* nascent transcripts (intron) and SCR protein, the detection scheme was as follows: *Scr*-cDNA- FITC>guinea pig anti-FITC>goat anti-guinea-pig Alexa594; *Scr*-intron- BIO>mouse anti-BIO>donkey anti-mouse Alexa488; rabbit anti-SCR>donkey anti-rabbit Alexa555.

To detect wildtype (*w^1118^*) expression patterns of *Antp* transcripts and *iab-4* transcripts, the sequential detection scheme was as follows: *Antp*-P1-BIO>mouse anti-BIO>donkey anti-mouse Alexa488; *iab-4-*DIG>sheep anti-DIG>donkey anti-sheep Alexa647. To detect wildtype (*w^1118^*) expression patterns of ANTP protein and *iab-4* transcripts the sequential detection scheme was mouse anti-ANTP>donkey anti-mouse Alexa488: *iab-4-*DIG>sheep anti-DIG>donkey anti-sheep Alexa647.

To visualize ANTP protein levels in *ΔmiR-iab-4/8*/*TM3,Ubx-LacZ* embryos, the detection scheme was as follows: *iab-4-*DNP>rabbit anti-DNP>donkey anti-rabbit Alexa555; mouse anti-ANTP>donkey anti-mouse Alexa488; *LacZ*-FITC>guinea pig anti-FITC>goat anti-guinea-pig Alexa594. *LacZ* staining was used to differentiate heterozygous and homozygous miRNA deletion embryos.

## Supporting Information

Figure S1 **Alignment of basal promoter regions of** ***pri-miR-10*** **from 12** ***Drosophila*** **species.** Pink shaded residues are not conserved with *D. melanogaster*. Highlighted in red, green and blue are promoter motifs Initiator (Inr), Motif Ten Element (MTE), and Downstream Promoter Element (DPE) respectively. Consensus sequences for motifs are given above alignment and differently shaded nucleotides are not conserved with *D. melanogaster*. *D. ana* - *Drosophila ananassae*, *D. ere* - *Drosophila erecta*, *D. gri* - *Drosophila grimshawi*, *D. mel* - *Drosophila melanogaster*, *D. mir* - *Drosophila miranda*, *D. moj* - *Drosophila mojavensis*, *D. per* - *Drosophila persimilis*, *D. pse* - *Drosophila pseudoobscura*, *D. sec* - *Drosophila sechellia*, *D. sim* - *Drosophila simulans*, *D. sub* - *Drosophila subobscura*, *D. vir* - *Drosophila virilus*, *D. wil* - *Drosophila willistoni*, *D. yak* - *Drosophila yakuba*.
(TIF)

Figure S2 **Alignment of** ***mir-10*** **hairpins from bilaterians.** Highlighted nucleotides are 100% conserved.
(TIF)

Figure S3 **Conservation of mature miRNA sequences in** ***mir-10*** **hairpins.** Highlighted and outlined sequences are 90% conserved in each alignment. (**A**) Both arms of arthropod *mir-10* hairpins which contribute to the Dicer cleavage product are conserved. (**B**) In deuterostomes the miR-10-5p sequence is conserved, while the 3p sequence is not. *Aaeg* - *Aedes aegypti*, *Abur* - *Astatotilapia burtoni*, *Acal* - *Amia calva*, *Acar* - *Anolis carolinensis*, *Acep* - *Atta cephalotes*, *Aflo* - *Apis florea*, *Afra* - *Allocentrotus fragilis*, *Agam* - *Anopheles gambiae*, *Amel* - *Apis mellifera*, *Apis* - *Acyrthosiphum pisum*, *Bflo* - *Branchiostoma floridae*, *Bmor* - *Bombyx mori*, *Btau* - *Bos Taurus*, *Bter* - *Bombus terrestris*, *Ccap* - *Ceratitis capitata*, *Cflo* - *Camponotus floridanus*, *Cmil* - *Callorhinchus milii*, *Cpip* - *Culex pipiens quinquefasciatus*, *Dana* - *Drosophila ananassae*, *Dere* - *Drosophila erecta*, *Dgri* - *Drosophila grimshawi*, *Dmel* - *Drosophila melanogaster*, *Dmir* - *Drosophila miranda*, *Dmoj* - *Drosophila mojavensis*, *Dper* - *Drosophila persimilis*, *Dpse* - *Drosophila pseudoobscura*, *Dpul* - *Daphnia pulex*, *Drer* - *Danio rerio*, *Dsec* - *Drosophila sechellia*, *Dsim* - *Drosophila simulans*, *Dsub* - *Drosophila subobscura*, *Dvir* - *Drosophila virilus*, *Dwil* - *Drosophila willistoni*, *Dyak* - *Drosophila yakuba*, *Ggal* - *Gallus gallus*, *Gmor* - *Glossina morsitans*, *Hsal* - *Harpegnathos saltator*, *Hsap* - *Homo sapiens*, *Isca* - *Ixodes scapularis, Lgig* - *Lottia gigantea*, *Llon* - *Lutzomyia longipalpis*, *Lmig* - *Locusta migratoria*, *Mdes* - *Mayetiola destructor*, *Mdom* - *Monodelphis domestica*, *Mmus* - *Mus musculus*, *Nlon* - *Nasonia longicornis*, *Nlug* - *Nilaparvata lugens*, *Nvit* - *Nasonia vitripennis*, *Oana* - *Ornithorhynchus anatinus*, *Phum* - *Pediculus Humanus*, *Ppap - Phlebotomus papatasi*, *Rpro* - *Rhodnius prolixus*, *Skow* - *Saccoglossus kowalevskii*, *Spur* - *Strongylocentrotus purpuratus*, *Tcas* - *Tribolium castaneum*, *Tgut* - *Taeniopygia guttata*, *Tnig* - *Tetraodon nigroviridis*, *Trub* - *Takifugu rubripes*, *Xtro* - *Xenopus tropicalis*.
(TIF)

Figure S4 **Schematic representations of Hox complexes and their miRNAs.** Transcriptional direction of Hox genes is from right to left in all cases. Hairpins on top are transcribed from right to left. Hairpins on bottom are transcribed from left to right. (**A**) Hox complex of the likely arthropod ancestor from comparison of sequenced species and locations of *mir-10*, *mir-993*, *mir-iab-4*, and *mir-iab-8* genes as hairpins. (**B**) Hox complex of the likely vertebrate ancestor from comparison of sequenced species and locations of *mir-10* and *mir-196* genes as hairpins. Additional posterior Hox genes not shown. (**C**) Hox complex of the likely Lophotrochozoan ancestor from comparison of miRNAs from *Capitella teleta* http://genome.jgi-psf.org/Capca1/Capca1.home.html, and the *Lottia gigantea* Hox complexes with locations of *mir-10*, *mir-993*, and a *mir-10-related* genes indicated as hairpins.
(TIF)

Figure S5 **Putative miR-10-5p target sites in the 3′UTRs of insect** ***Sex combs reduced*** **(** ***Scr*** **) orthologs are conserved in regions of relatively poor conservation.** Alignment of conserved sequences found in the 3′UTRs (or 3′ of the stop codon in putative UTR sequence) of *Scr* genes in insects and complementarity to mature miR-10-5p sequence. Highlighted nucleotides are 60% conserved. Outlined in red are nucleotides that can pair with miR-10-5p. *Aaeg* - *Aedes aegypti*, *Bter* - *Bombus terrestris*, *Cflo* - *Camponotus floridanus*, *Cpip* - *Culex pipiens quinquefasciatus*, *Dana* - *Drosophila ananassae*, *Dere* - *Drosophila erecta*, *Dgri* - *Drosophila grimshawi*, *Dmel* - *Drosophila melanogaster*, *Dmir* - *Drosophila miranda*, *Dmoj* - *Drosophila mojavensis*, *Dper* - *Drosophila persimilis*, *Dpse* - *Drosophila pseudoobscura*, *Dsec* - *Drosophila sechellia*, *Dsim* - *Drosophila simulans*, *Dsub* - *Drosophila subobscura*, *Dvir* - *Drosophila virilus*, *Dwil* - *Drosophila willistoni*, *Dyak* - *Drosophila yakuba*, *Gmor* - *Glossina morsitans*, *Hsal* - *Harpegnathos saltator*, *Isca* - *Ixodes scapularis, Llon* - *Lutzomyia longipalpis*, *Mdes* - *Mayetiola destructor*, *Nvit* - *Nasonia vitripennis*, *Phum* - *Pediculus Humanus*, *Ppap - Phlebotomus papatasi*, *Tcas* - *Tribolium castaneum*.
(TIF)

Figure S6 **Putative miR-10-3p target sites in the 3′UTRs of** ***Brachyceran Abdominal-B*** **(** ***Abd-B*** **) orthologs are conserved in regions of relatively poor conservation.** Alignment of conserved sequences found in the 3′UTRs (or 3′ of the stop codon in putative UTR sequence) of *Abd-B* genes in *Brachycerans* and complementarity to mature miR-10-3p sequence. Highlighted nucleotides are 85% conserved. Outlined in red are nucleotides which can pair with miR-10-3p. *Dana* - *Drosophila ananassae*, *Dere* - *Drosophila erecta*, *Dgri* - *Drosophila grimshawi*, *Dmel* - *Drosophila melanogaster*, *Dmir* - *Drosophila miranda*, *Dmoj* - *Drosophila mojavensis*, *Dper* - *Drosophila persimilis*, *Dpse* - *Drosophila pseudoobscura*, *Dsec* - *Drosophila sechellia*, *Dsim* - *Drosophila simulans*, *Dsub* - *Drosophila subobscura*, *Dvir* - *Drosophila virilus*, *Dwil* - *Drosophila willistoni*, *Dyak* - *Drosophila yakuba, Ccap* - *Ceratitis capitata*, *Gmor* - *Glossina morsitans.*
(TIF)

Figure S7 ***Scr*** **protein and cytoplasmic transcript accumulation are downregulated in the ventral first thoracic segment.** (**A**) The pattern of *Scr* cytoplasmic transcript accumulation in the ectoderm includes all of the labial segment as well as lateral regions of the first thoracic segment. The cells which are transcribing *Scr*, seen with intron probe – green in (**B**), include all of the ventral and lateral domains of both the labial segment and the first thoracic segment. SCR protein accumulation does not occur in all cells which are transcribing *Scr* (**C**) but coincides completely with those cells that show high levels of cytoplasmic transcript accumulation (**D**).
(TIF)

Figure S8 **Mature miRNA is produced from transgenes and results in aberrant phenotypes.** When expressed from a *prd*-GAL4 driver (prd>*pri-mir-10*), cytoplasmic miR-10-5p is detectable in the *prd* pattern at high levels in a germband extended embryo (**A**). When expressed from an Actin-GAL4 (Act5C>*pri-mir-10*) (**C**) or *da*-GAL4 driver (da>*pri-mir-10*) (**D**) malformations are noticeable in the head cuticle (arrows) when compared to wild type (**B**). Two small black dots (sensory organs) can be seen in the dorsal anterior head in some cuticular preparations, but not others. This is not because of an actual sensory organ duplication, but due to differential flattening of the cuticles during their preparation, so that in some cuticles the sensory organs from both sides of the anterior dorsal head can be seen.
(TIF)

Figure S9 **Putative miR-iab-4-5p target sites in the 3′UTRs of** ***Brachyceran Antennapedia*** **(** ***Antp*** **) orthologs are conserved in regions of relatively poor conservation.** Alignment of conserved sequences found in the 3′UTRs (or 3′ of the stop codon in putative UTR sequence) of *Antp* genes in *Brachycerans* and complementarity to mature miR-iab-4-5p sequence. Highlighted nucleotides are 90% conserved. Outlined in red are nucleotides which can pair with miR-iab-4-5p. *Dana* - *Drosophila ananassae*, *Dere* - *Drosophila erecta*, *Dgri* - *Drosophila grimshawi*, *Dmel* - *Drosophila melanogaster*, *Dmir* - *Drosophila miranda*, *Dmoj* - *Drosophila mojavensis*, *Dper* - *Drosophila persimilis*, *Dpse* - *Drosophila pseudoobscura*, *Dsec* - *Drosophila sechellia*, *Dsim* - *Drosophila simulans*, *Dsub* - *Drosophila subobscura*, *Dvir* - *Drosophila virilus*, *Dwil* - *Drosophila willistoni*, *Dyak* - *Drosophila yakuba, Mdes* - *Mayetiola destructor*.
(TIF)
