# Supplementary figures and images for: Three Drosophila Hox Complex microRNAs Do Not Have Major Effects on Expression of Evolutionarily Conserved Hox Gene Targets during Embryogenesis
Source: PLoS One. 2012 Feb 29;7(2):e31365. doi: 10.1371/journal.pone.0031365 (PMC3290615; doi:10.1371/journal.pone.0031365)

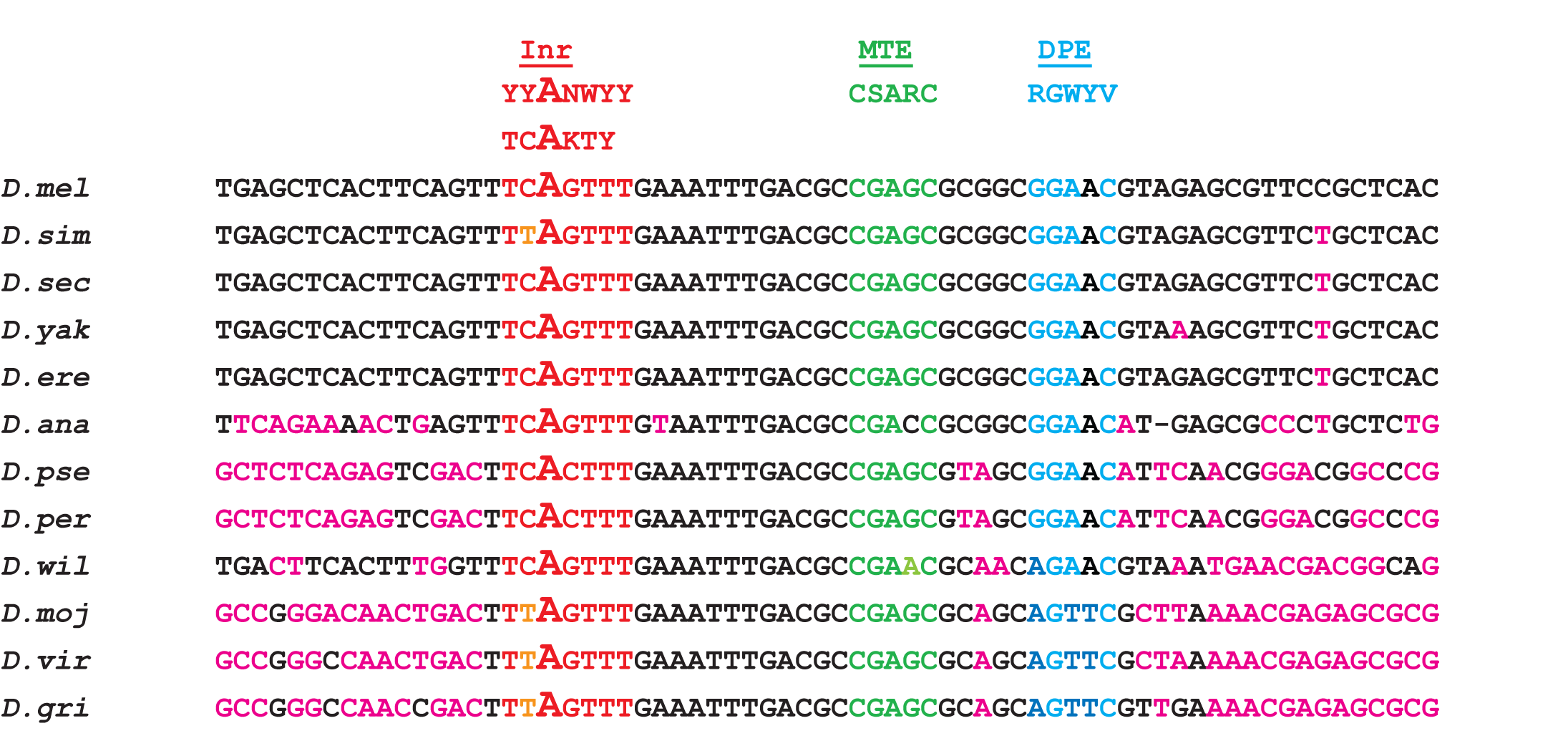

Supplement: Figure S1 — Alignment of basal promoter regions of pri-miR-10 from 12 Drosophila species. Pink shaded residues are not conserved with D. melanogaster. Highlighted in red, green and blue are promoter motifs Initiator (Inr), Motif Ten Element (MTE), and Downstream Promoter Element (DPE) respectively. Consensus sequences for motifs are given above alignment and differently shaded nucleotides are not conserved with D. melanogaster. D. ana - Drosophila ananassae, D. ere - Drosophila erecta, D. gri - Drosophila grimshawi, D. mel - Drosophila melanogaster, D. mir - Drosophila miranda, D. moj - Drosophila mojavensis, D. per - Drosophila persimilis, D. pse - Drosophila pseudoobscura, D. sec - Drosophila sechellia, D. sim - Drosophila simulans, D. sub - Drosophila subobscura, D. vir - Drosophila virilus, D. wil - Drosophila willistoni, D. yak - Drosophila yakuba. (TIF) [file pone.0031365.s001.tif]

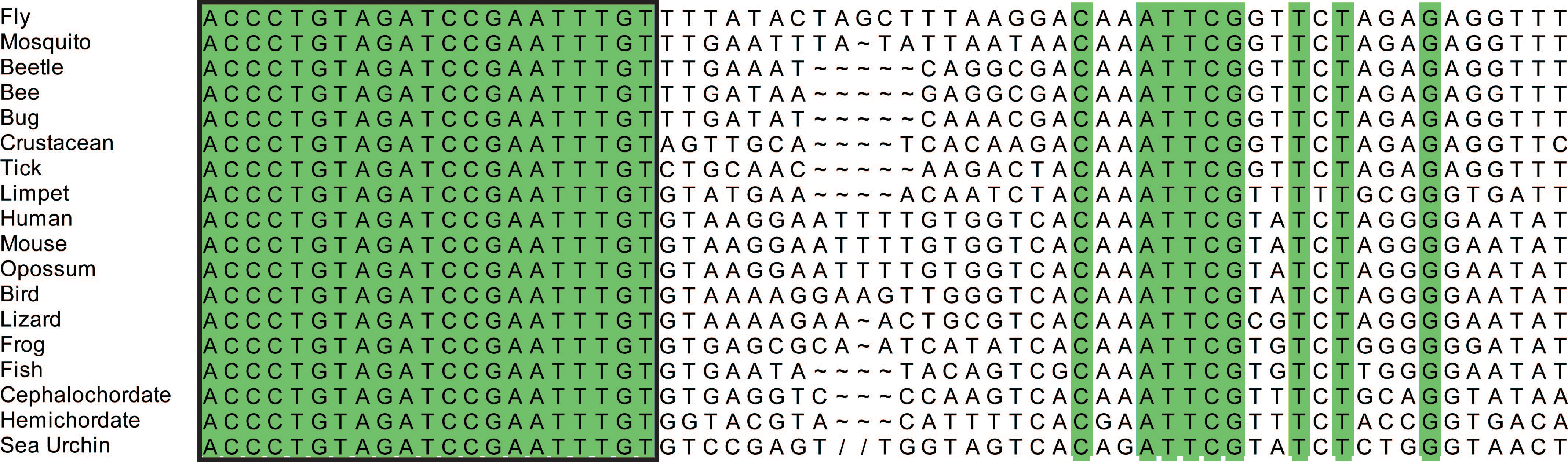

Supplement: Figure S2 — Alignment of mir-10 hairpins from bilaterians. Highlighted nucleotides are 100% conserved. (TIF) [file pone.0031365.s002.tif]

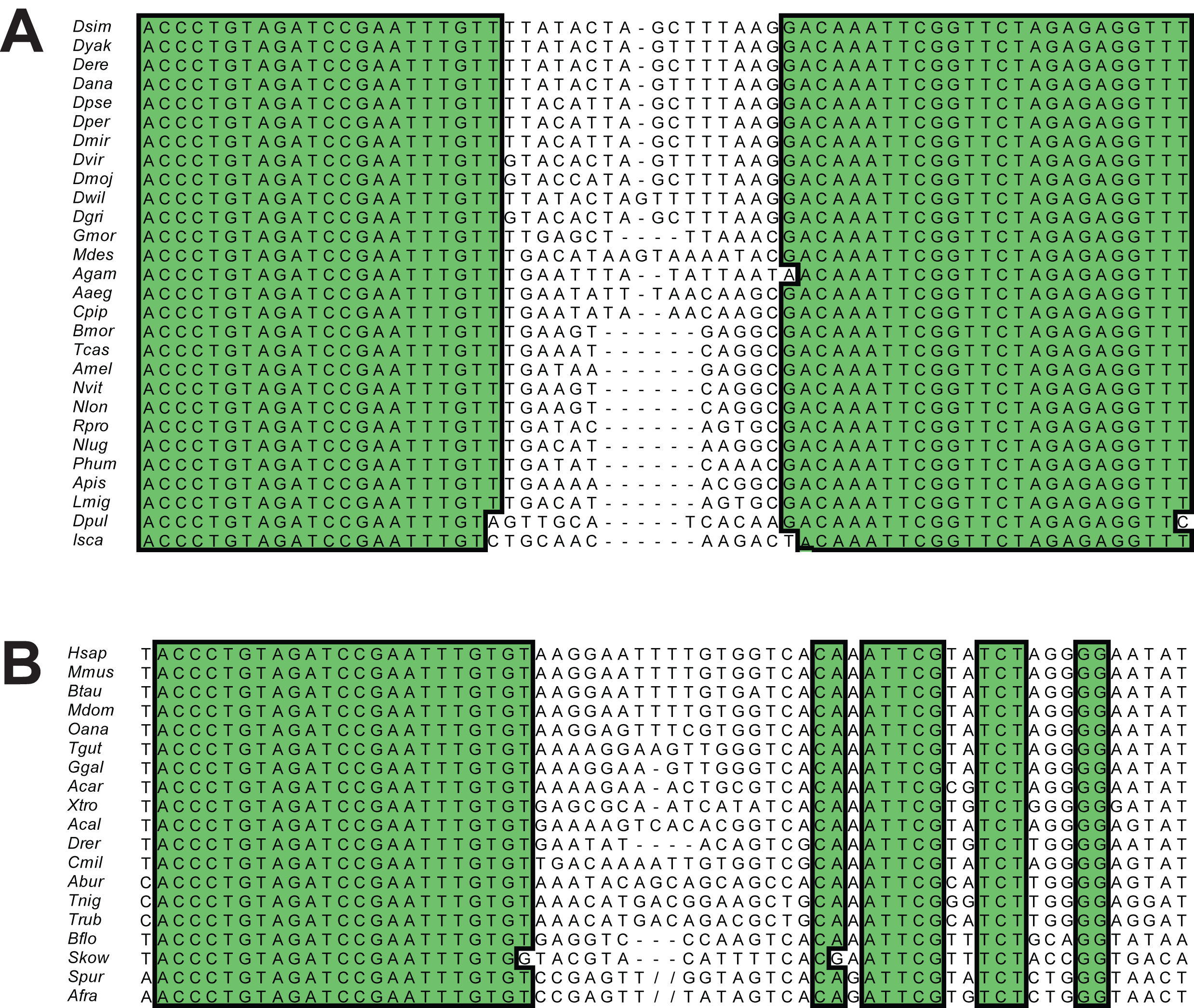

Supplement: Figure S3 — Conservation of mature miRNA sequences in mir-10 hairpins. Highlighted and outlined sequences are 90% conserved in each alignment. (A) Both arms of arthropod mir-10 hairpins which contribute to the Dicer cleavage product are conserved. (B) In deuterostomes the miR-10-5p sequence is conserved, while the 3p sequence is not. Aaeg - Aedes aegypti, Abur - Astatotilapia burtoni, Acal - Amia calva, Acar - Anolis carolinensis, Acep - Atta cephalotes, Aflo - Apis florea, Afra - Allocentrotus fragilis, Agam - Anopheles gambiae, Amel - Apis mellifera, Apis - Acyrthosiphum pisum, Bflo - Branchiostoma floridae, Bmor - Bombyx mori, Btau - Bos Taurus, Bter - Bombus terrestris, Ccap - Ceratitis capitata, Cflo - Camponotus floridanus, Cmil - Callorhinchus milii, Cpip - Culex pipiens quinquefasciatus, Dana - Drosophila ananassae, Dere - Drosophila erecta, Dgri - Drosophila grimshawi, Dmel - Drosophila melanogaster, Dmir - Drosophila miranda, Dmoj - Drosophila mojavensis, Dper - Drosophila persimilis, Dpse - Drosophila pseudoobscura, Dpul - Daphnia pulex, Drer - Danio rerio, Dsec - Drosophila sechellia, Dsim - Drosophila simulans, Dsub - Drosophila subobscura, Dvir - Drosophila virilus, Dwil - Drosophila willistoni, Dyak - Drosophila yakuba, Ggal - Gallus gallus, Gmor - Glossina morsitans, Hsal - Harpegnathos saltator, Hsap - Homo sapiens, Isca - Ixodes scapularis, Lgig - Lottia gigantea, Llon - Lutzomyia longipalpis, Lmig - Locusta migratoria, Mdes - Mayetiola destructor, Mdom - Monodelphis domestica, Mmus - Mus musculus, Nlon - Nasonia longicornis, Nlug - Nilaparvata lugens, Nvit - Nasonia vitripennis, Oana - Ornithorhynchus anatinus, Phum - Pediculus Humanus, Ppap - Phlebotomus papatasi, Rpro - Rhodnius prolixus, Skow - Saccoglossus kowalevskii, Spur - Strongylocentrotus purpuratus, Tcas - Tribolium castaneum, Tgut - Taeniopygia guttata, Tnig - Tetraodon nigroviridis, Trub - Takifugu rubripes, Xtro - Xenopus tropicalis. (TIF) [file pone.0031365.s003.tif]

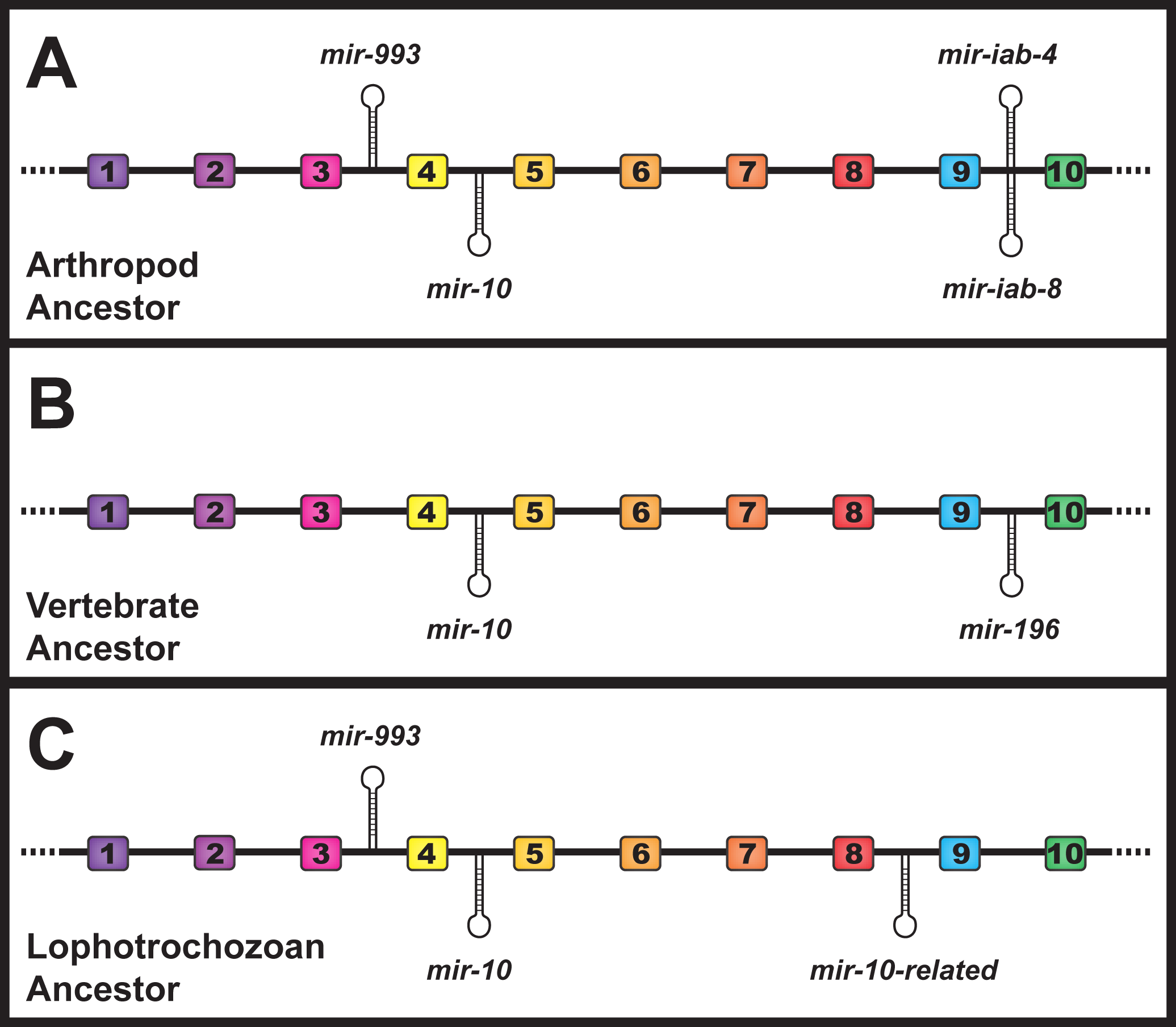

Supplement: Figure S4 — Schematic representations of Hox complexes and their miRNAs. Transcriptional direction of Hox genes is from right to left in all cases. Hairpins on top are transcribed from right to left. Hairpins on bottom are transcribed from left to right. (A) Hox complex of the likely arthropod ancestor from comparison of sequenced species and locations of mir-10, mir-993, mir-iab-4, and mir-iab-8 genes as hairpins. (B) Hox complex of the likely vertebrate ancestor from comparison of sequenced species and locations of mir-10 and mir-196 genes as hairpins. Additional posterior Hox genes not shown. (C) Hox complex of the likely Lophotrochozoan ancestor from comparison of miRNAs from Capitella teleta http://genome.jgi-psf.org/Capca1/Capca1.home.html, and the Lottia gigantea Hox complexes with locations of mir-10, mir-993, and a mir-10-related genes indicated as hairpins. (TIF) [file pone.0031365.s004.tif]

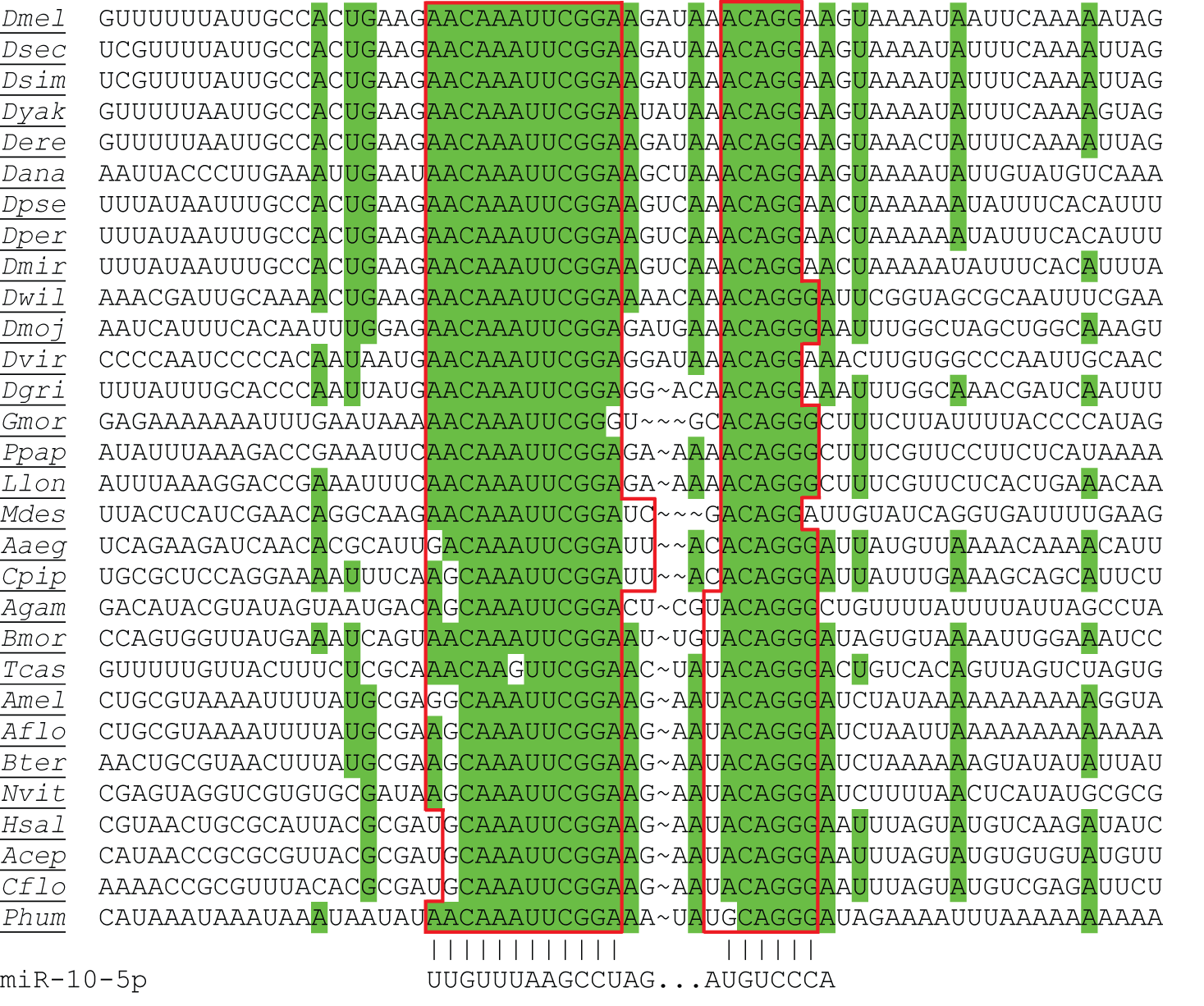

Supplement: Figure S5 — Putative miR-10-5p target sites in the 3′UTRs of insect Sex combs reduced ( Scr ) orthologs are conserved in regions of relatively poor conservation. Alignment of conserved sequences found in the 3′UTRs (or 3′ of the stop codon in putative UTR sequence) of Scr genes in insects and complementarity to mature miR-10-5p sequence. Highlighted nucleotides are 60% conserved. Outlined in red are nucleotides that can pair with miR-10-5p. Aaeg - Aedes aegypti, Bter - Bombus terrestris, Cflo - Camponotus floridanus, Cpip - Culex pipiens quinquefasciatus, Dana - Drosophila ananassae, Dere - Drosophila erecta, Dgri - Drosophila grimshawi, Dmel - Drosophila melanogaster, Dmir - Drosophila miranda, Dmoj - Drosophila mojavensis, Dper - Drosophila persimilis, Dpse - Drosophila pseudoobscura, Dsec - Drosophila sechellia, Dsim - Drosophila simulans, Dsub - Drosophila subobscura, Dvir - Drosophila virilus, Dwil - Drosophila willistoni, Dyak - Drosophila yakuba, Gmor - Glossina morsitans, Hsal - Harpegnathos saltator, Isca - Ixodes scapularis, Llon - Lutzomyia longipalpis, Mdes - Mayetiola destructor, Nvit - Nasonia vitripennis, Phum - Pediculus Humanus, Ppap - Phlebotomus papatasi, Tcas - Tribolium castaneum. (TIF) [file pone.0031365.s005.tif]

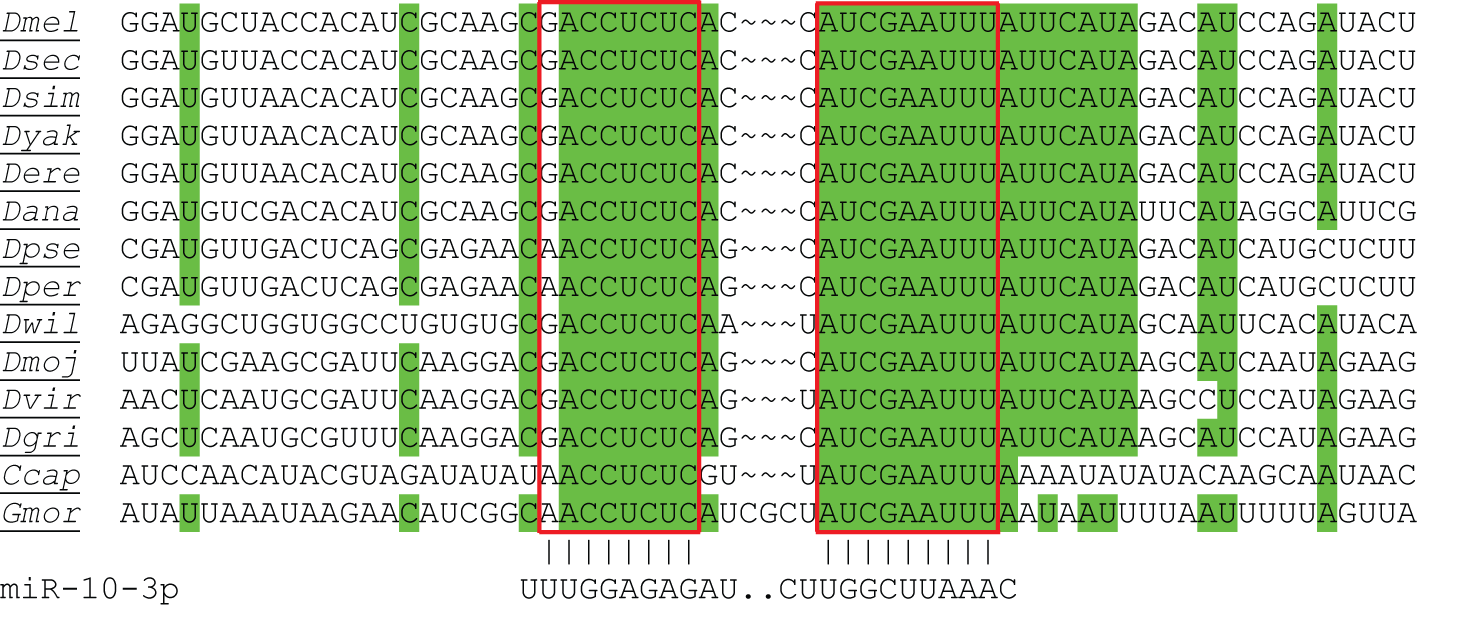

Supplement: Figure S6 — Putative miR-10-3p target sites in the 3′UTRs of Brachyceran Abdominal-B ( Abd-B ) orthologs are conserved in regions of relatively poor conservation. Alignment of conserved sequences found in the 3′UTRs (or 3′ of the stop codon in putative UTR sequence) of Abd-B genes in Brachycerans and complementarity to mature miR-10-3p sequence. Highlighted nucleotides are 85% conserved. Outlined in red are nucleotides which can pair with miR-10-3p. Dana - Drosophila ananassae, Dere - Drosophila erecta, Dgri - Drosophila grimshawi, Dmel - Drosophila melanogaster, Dmir - Drosophila miranda, Dmoj - Drosophila mojavensis, Dper - Drosophila persimilis, Dpse - Drosophila pseudoobscura, Dsec - Drosophila sechellia, Dsim - Drosophila simulans, Dsub - Drosophila subobscura, Dvir - Drosophila virilus, Dwil - Drosophila willistoni, Dyak - Drosophila yakuba, Ccap - Ceratitis capitata, Gmor - Glossina morsitans. (TIF) [file pone.0031365.s006.tif]

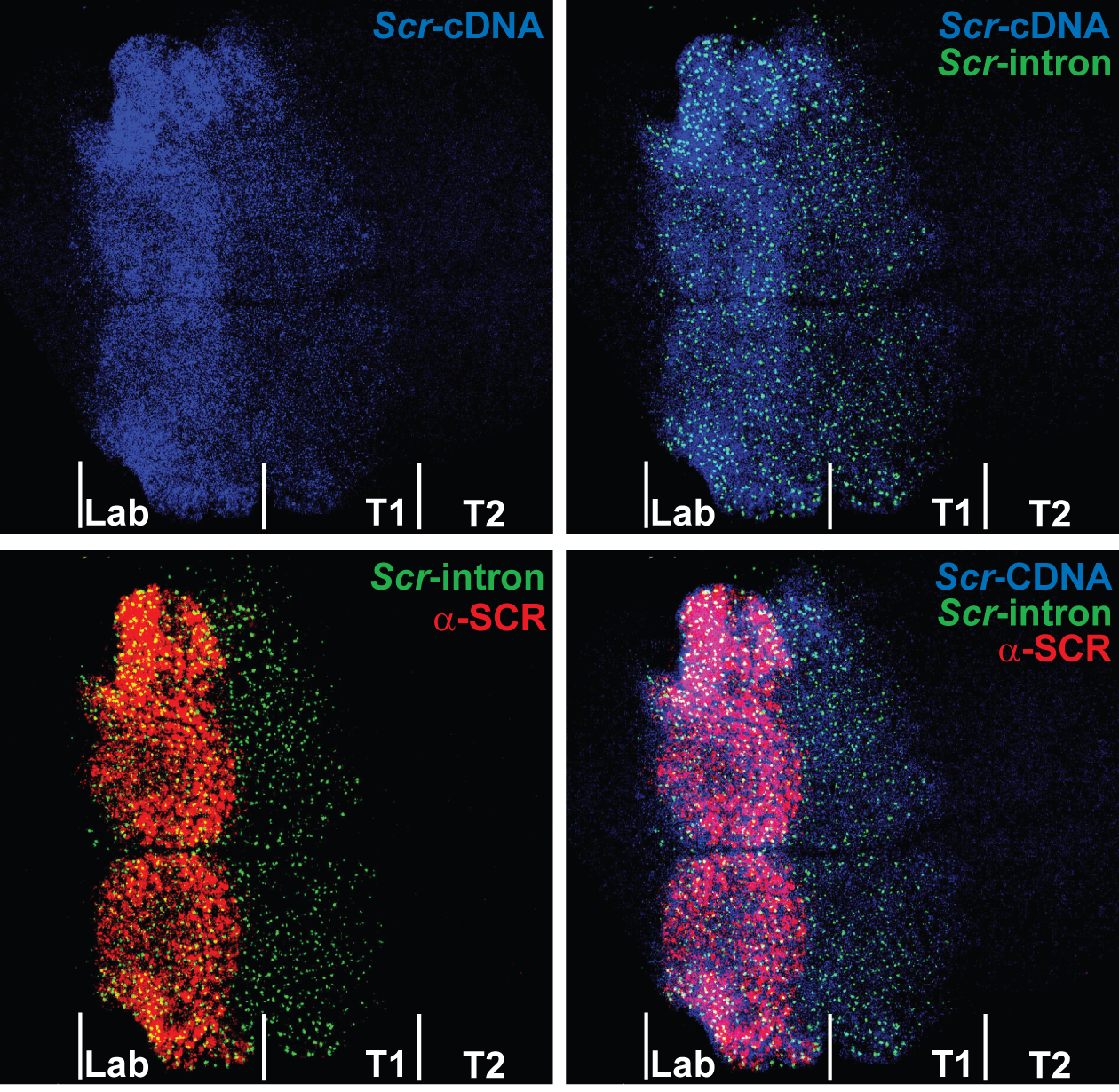

Supplement: Figure S7 — Scr protein and cytoplasmic transcript accumulation are downregulated in the ventral first thoracic segment. (A) The pattern of Scr cytoplasmic transcript accumulation in the ectoderm includes all of the labial segment as well as lateral regions of the first thoracic segment. The cells which are transcribing Scr, seen with intron probe – green in (B), include all of the ventral and lateral domains of both the labial segment and the first thoracic segment. SCR protein accumulation does not occur in all cells which are transcribing Scr (C) but coincides completely with those cells that show high levels of cytoplasmic transcript accumulation (D). (TIF) [file pone.0031365.s007.tif]

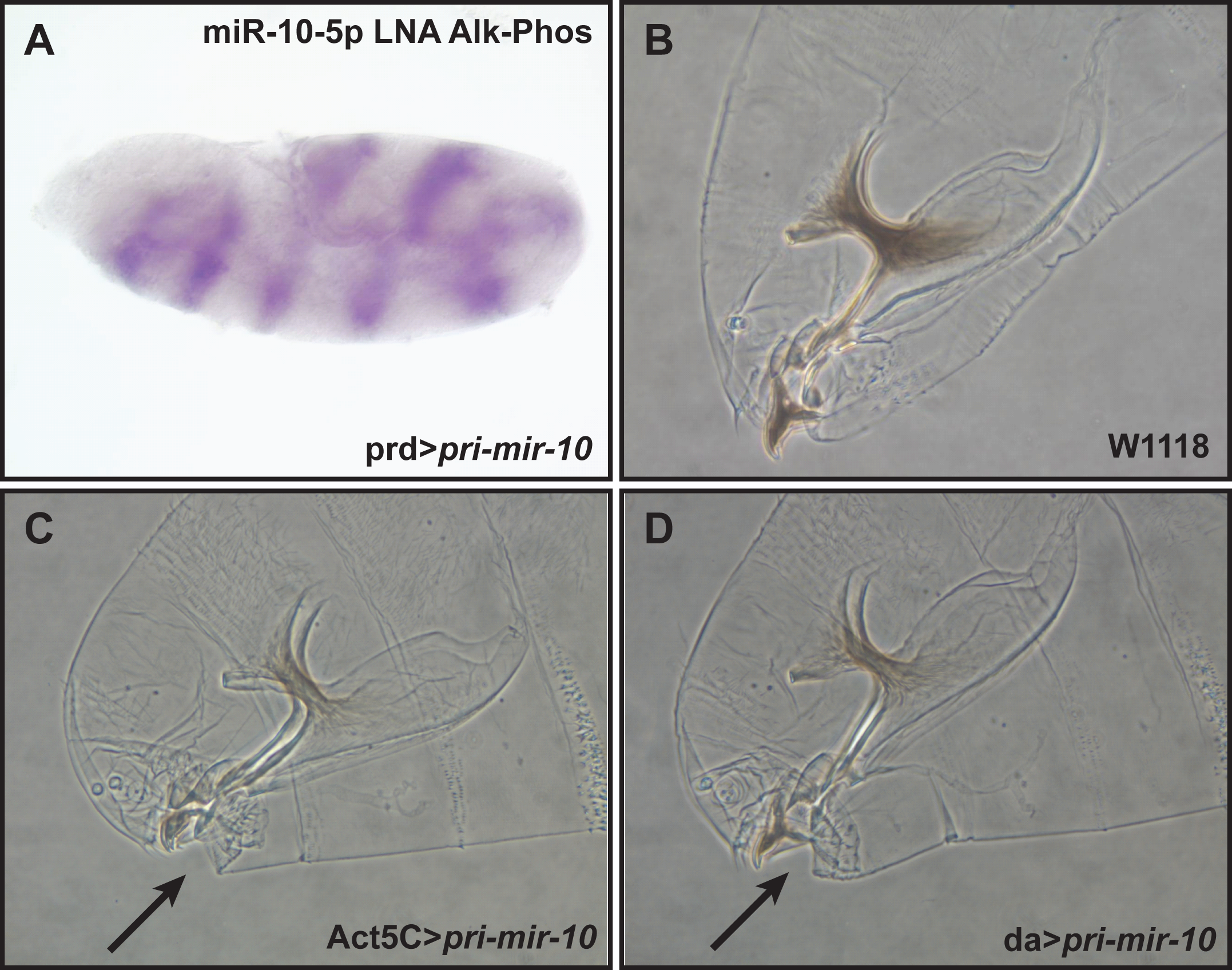

Supplement: Figure S8 — Mature miRNA is produced from transgenes and results in aberrant phenotypes. When expressed from a prd-GAL4 driver (prd>pri-mir-10), cytoplasmic miR-10-5p is detectable in the prd pattern at high levels in a germband extended embryo (A). When expressed from an Actin-GAL4 (Act5C>pri-mir-10) (C) or da-GAL4 driver (da>pri-mir-10) (D) malformations are noticeable in the head cuticle (arrows) when compared to wild type (B). Two small black dots (sensory organs) can be seen in the dorsal anterior head in some cuticular preparations, but not others. This is not because of an actual sensory organ duplication, but due to differential flattening of the cuticles during their preparation, so that in some cuticles the sensory organs from both sides of the anterior dorsal head can be seen. (TIF) [file pone.0031365.s008.tif]

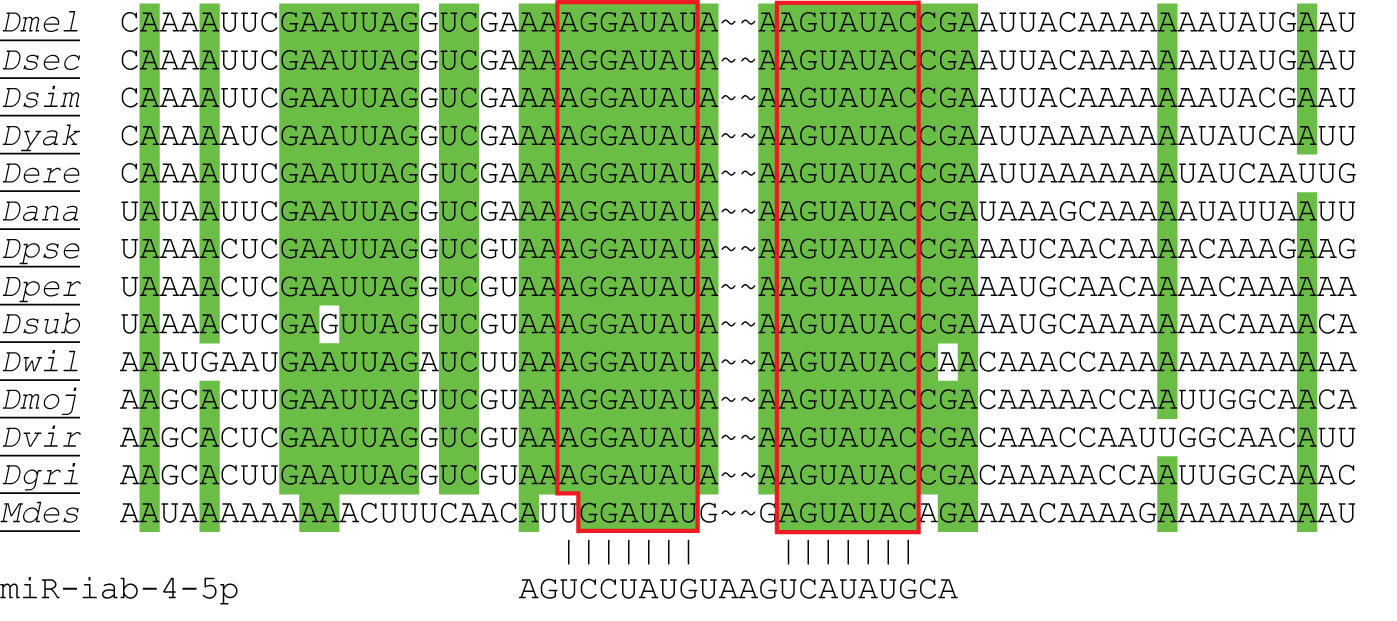

Supplement: Figure S9 — Putative miR-iab-4-5p target sites in the 3′UTRs of Brachyceran Antennapedia ( Antp ) orthologs are conserved in regions of relatively poor conservation. Alignment of conserved sequences found in the 3′UTRs (or 3′ of the stop codon in putative UTR sequence) of Antp genes in Brachycerans and complementarity to mature miR-iab-4-5p sequence. Highlighted nucleotides are 90% conserved. Outlined in red are nucleotides which can pair with miR-iab-4-5p. Dana - Drosophila ananassae, Dere - Drosophila erecta, Dgri - Drosophila grimshawi, Dmel - Drosophila melanogaster, Dmir - Drosophila miranda, Dmoj - Drosophila mojavensis, Dper - Drosophila persimilis, Dpse - Drosophila pseudoobscura, Dsec - Drosophila sechellia, Dsim - Drosophila simulans, Dsub - Drosophila subobscura, Dvir - Drosophila virilus, Dwil - Drosophila willistoni, Dyak - Drosophila yakuba, Mdes - Mayetiola destructor. (TIF) [file pone.0031365.s009.tif]
